# Avian Test Battery for the Evaluation of Developmental Abnormalities of Neuro- and Reproductive Systems

**DOI:** 10.3389/fnins.2016.00296

**Published:** 2016-06-30

**Authors:** Takaharu Kawashima, Walaa M. S. Ahmed, Koki Nagino, Takayoshi Ubuka, Kazuyoshi Tsutsui

**Affiliations:** ^1^Center for Environmental Biology and Ecosystem Studies, National Institute for Environmental StudiesTsukuba, Japan; ^2^Center for Environmental Health Sciences, National Institute for Environmental StudiesTsukuba, Japan; ^3^Department of Clinical Pathology, Faculty of Veterinary Medicine, Beni-Suef UniversityBeni-Suef, Egypt; ^4^Laboratory of Integrative Brain Sciences, Department of Biology and Center for Medical Life Science, Waseda UniversityTokyo, Japan; ^5^Jeffrey Cheah School of Medicine and Health Sciences, Brain Research Institute Monash Sunway, Monash University MalaysiaBandar Sunway, Malaysia

**Keywords:** embryo culture system, sociosexual behavior, imprinting behavior, developmental neurotoxicity, Galliformes

## Abstract

Most of the currently used toxicity assays for environmental chemicals use acute or chronic systemic or reproductive toxicity endpoints rather than neurobehavioral endpoints. In addition, the current standard approaches to assess reproductive toxicity are time-consuming. Therefore, with increasing numbers of chemicals being developed with potentially harmful neurobehavioral effects in higher vertebrates, including humans, more efficient means of assessing neuro- and reproductive toxicity are required. Here we discuss the use of a Galliformes-based avian test battery in which developmental toxicity is assessed by means of a combination of chemical exposure during early embryonic development using an embryo culture system followed by analyses after hatching of sociosexual behaviors such as aggression and mating and of visual memory via filial imprinting. This Galliformes-based avian test battery shows promise as a sophisticated means not only of assessing chemical toxicity in avian species but also of assessing the risks posed to higher vertebrates, including humans, which are markedly sensitive to nervous or neuroendocrine system dysfunction.

## Introduction

Avian experimental models are an important tool for elucidating fundamental principles in research fields such as embryology, endocrinology, genetics, neurology, and ethology (Le Douarin, 2004; Stern, 2005; Emery, 2006; Nakamori et al., 2013). Since the embryos of common Galliformes such as the chicken *(Gallus gallus domesticus*) and Japanese quail (*Coturnix japonica*) can easily be observed and directly manipulated during embryogenesis, they have become the model organisms most widely used in developmental biology (Le Douarin and Dieterlen-Lièvre, 2013; Nakamura and Funahashi, 2013; Sato, 2013; Suzuki, 2013). Recently, Japanese quail has also been used as a model organism in neurophysiological and neuroendocrine studies of sociosexual behavior (Ubuka et al., 2013; Ubuka and Tsutsui, 2014).

Recently, it has been suggested that similarities exist between mammals and birds in the sex differentiation of core sexual behaviors that is induced by gonadal hormones during embryonic development (Maekawa et al., 2014). Furthermore, Clayton and Emery (2015) have proposed that avian experimental models for human cognition could be adapted for studying the neural basis of complex cognition; reasoning, e.g., mean flexibility, problem solving, prospection, and declarative knowledge, and understanding the evolution and neurobiology of cognition, e.g. specific cognitive functions and critical roles of the avian and mammalian brain. Thus, avian experimental models represent a potentially powerful platform for elucidating the developmental mechanisms of the nervous and reproductive systems in higher vertebrates, including humans.

The negative effects of endocrine-disrupting chemicals, such as pesticides and herbicides, on neurodevelopmental processes and reproductive functions in wildlife and humans have been known for over a quarter of a century (Colborn et al., 1993; Lewis and Ford, 2012). Recently, house dust and flame retardants have also been identified as endocrine-disrupting chemicals (Suzuki et al., 2013). It has been proposed that endocrine-disrupting chemicals exert greater toxic effects during periods when organisms are more sensitive to hormonal disruption, such as during the intrauterine, perinatal, and juvenile periods and during puberty (Frye et al., 2012).

The existing developmental neurotoxicity tests have been carried out for some chemicals such as pesticides and their effects have been studied. However, there are problems to correspond to the large number of new chemicals because of high costs, long test period and high numbers of pregnant laboratory mammals used. Moreover, for inspection of embryonic development, the mothers have to be sacrificed which are of ethical concern (Lu et al., 2014). Therefore, simpler and alternative methods are desired. Avian culture techniques using embryonic tissues may offer certain advantages over *in vivo* experiments especially when developmental toxicity is anticipated (Neubert, 1982). The avian models are advantageous at three points in comparison to the conventional mammalian model for the assessment of the developmental toxicity: (1) direct manipulation, (2) continuous observation, and (3) reduction of unnecessary sacrifices of the pregnant individuals. In conventional developmental toxicity studies, the toxic effects of test compounds are examined in fetuses by using pregnant model organisms, such as rats and mice. However, avian embryos may be a better platform than mammalian embryos because the former can be observed and manipulated directly. Moreover, innovative avian embryo culture systems (ECSs) now allow continuous quantitative observations after administration of the test compound (Perry, 1988; Kawashima et al., 2005). Avian models also have ethical advantages because mammalian toxicity tests usually require sacrifice of the pregnant animals prior to examination of embryonic development, whereas avian models do not.

To allow more detailed evaluation of the neuro- and reproductive toxicities of environmental chemicals, new neurobehavioral endpoints in avian test models, such as sex differentiation in the gonads and brain, need to be established. Here we discuss the usefulness of an avian test battery with Galliformes for the assessment of developmental toxicity by using a combination of chemical exposure during early embryonic development by using an ECS followed by analyses after hatching of sociosexual behaviors such as aggression and mating and of visual memory via filial imprinting. Although, avian model systems are already commonly used in basic research (Le Douarin and Dieterlen-Lièvre, 2013; Nakamura and Funahashi, 2013), this article presents one of most alternative solutions as the evaluation of developmental abnormalities of neuro- and reproductive systems.

## Avian bioresources

**Figure 1:**
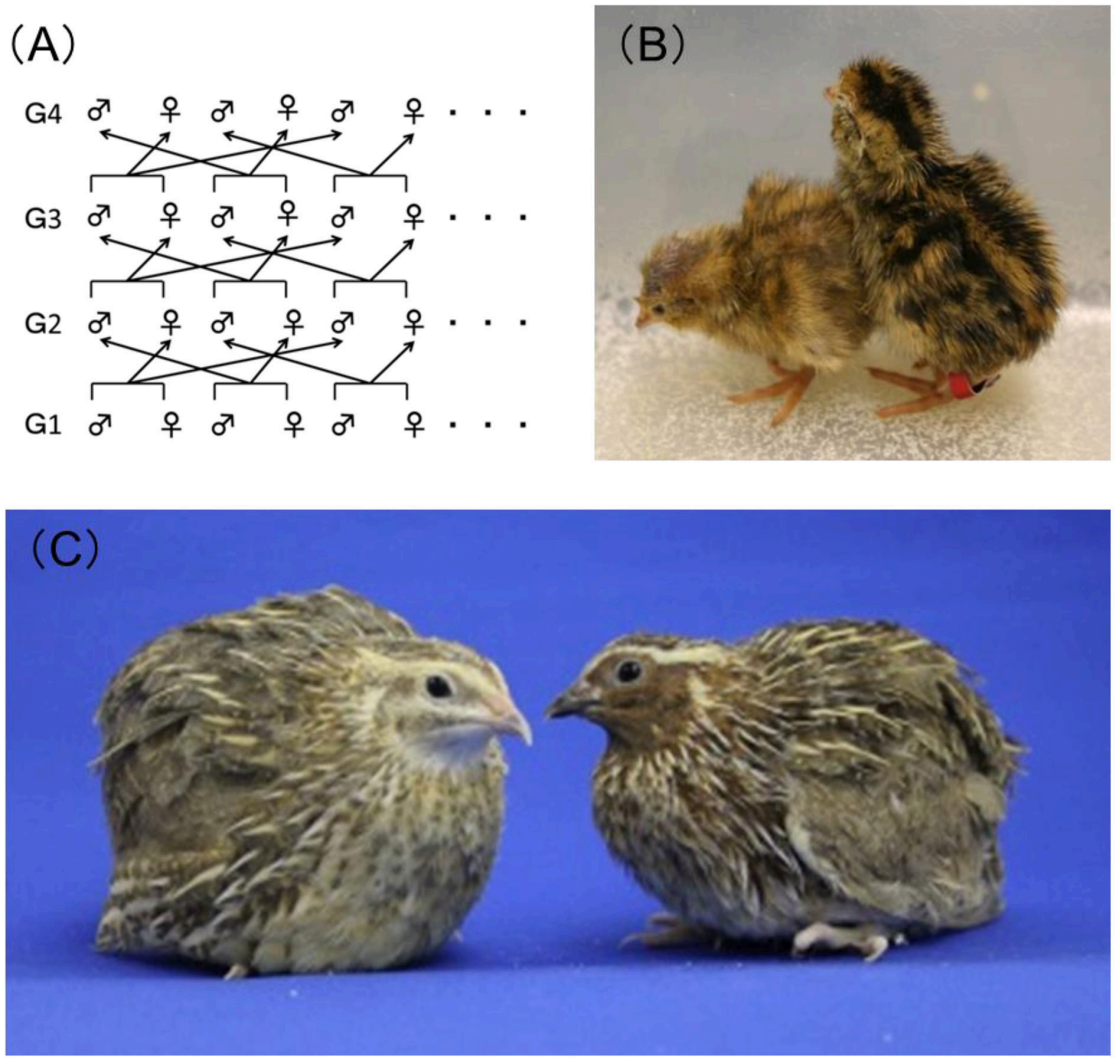
**Closed colony of Japanese quail at the National Institute for Environmental Studies, Japan. (A)** Overview of the rotational crossbreeding of the NIES-L quail strain from generations 1 (G1)–4 (G4). **(B)** Newly hatched NIES-L quail chicks with the yellow-brown plumage color phenotype (left) and the wild-type phenotype (right). **(C)** Male (right) and female (left) adult NIES-L quails.

Genetically homogeneous inbred strains of rats or mice are often used in toxicity studies to ensure reproducibility of the experimental results. However, no fully genetically inbred avian strains are currently available. It has now been more than half a century since the Japanese quail was evaluated and recommended as a laboratory animal by Padgett and Ivey (1959), and since then the Japanese quail has become of high value to researchers, especially those in the fields of embryology and physiology, because of its hardiness, ease of handling, precocity, and high egg productivity. In 1980, our group at the National Institute for Environmental Studies (NIES), Japan, began developing a closed colony of Japanese quail with the goal of establishing a new experimental model organism. This Japanese quail is maintained by means of rotational crossbreeding (Figure 1A) and is fixed with a yellow-brown plumage color mutation (Figures 1B,C). This strain is named NIES-L as mentioned below.

Recently, a draft genome sequence of the NIES-L quail was produced by means of next-generation sequencing, and 100 microsatellite markers have been developed as useful tools for evaluating the genetic variation within and between quail populations (Kawahara-Miki et al., 2013). Tadano et al. (2014) compared the estimated level of inbreeding within the NIES-L colony with that of a commercial random-bred colony by using polymorphic microsatellite marker analyses and confirmed that the heterozygosity in the NIES-L colony is gradually being lost over time. Our group is also using specific alleles of the microsatellite loci to monitor for genetic contamination in the colony by genetic markers developed. The Japanese quail is a potentially useful experimental organism not only for studies in the field of poultry science but also in the field of basic research and/or environmental studies, which are more necessary to analyze genetically.

About 35 years ago, we started to select and breed Japanese quails for high (H) and low (i.e., NIES-L) serum antibody titers against inactivated Newcastle disease virus (Takahashi et al., 1984), but the H quails have been extinct. It is clear that heterozygosity in the NIES-L closed colony is being gradually lost over time. However, it is difficult to establish mutant quail strains and maintain inbred strains because they are particularly susceptible to inbreeding depression (Sittmann et al., 1966). Indeed, no inbred strain of Japanese quail surviving more than 20 consecutive generations of full-sib mating has ever been developed, unlike in mouse and rat. Therefore, if we could fully establish an inbred strain of Japanese quail by continuing our breeding of NIES-L, this model organism would be useful for toxicity testing and would provide a simple and reproducible platform for the assessment of nervous and reproductive system developmental toxicity in birds and mammals.

## Avian embryo culture system

**Figure 2:**
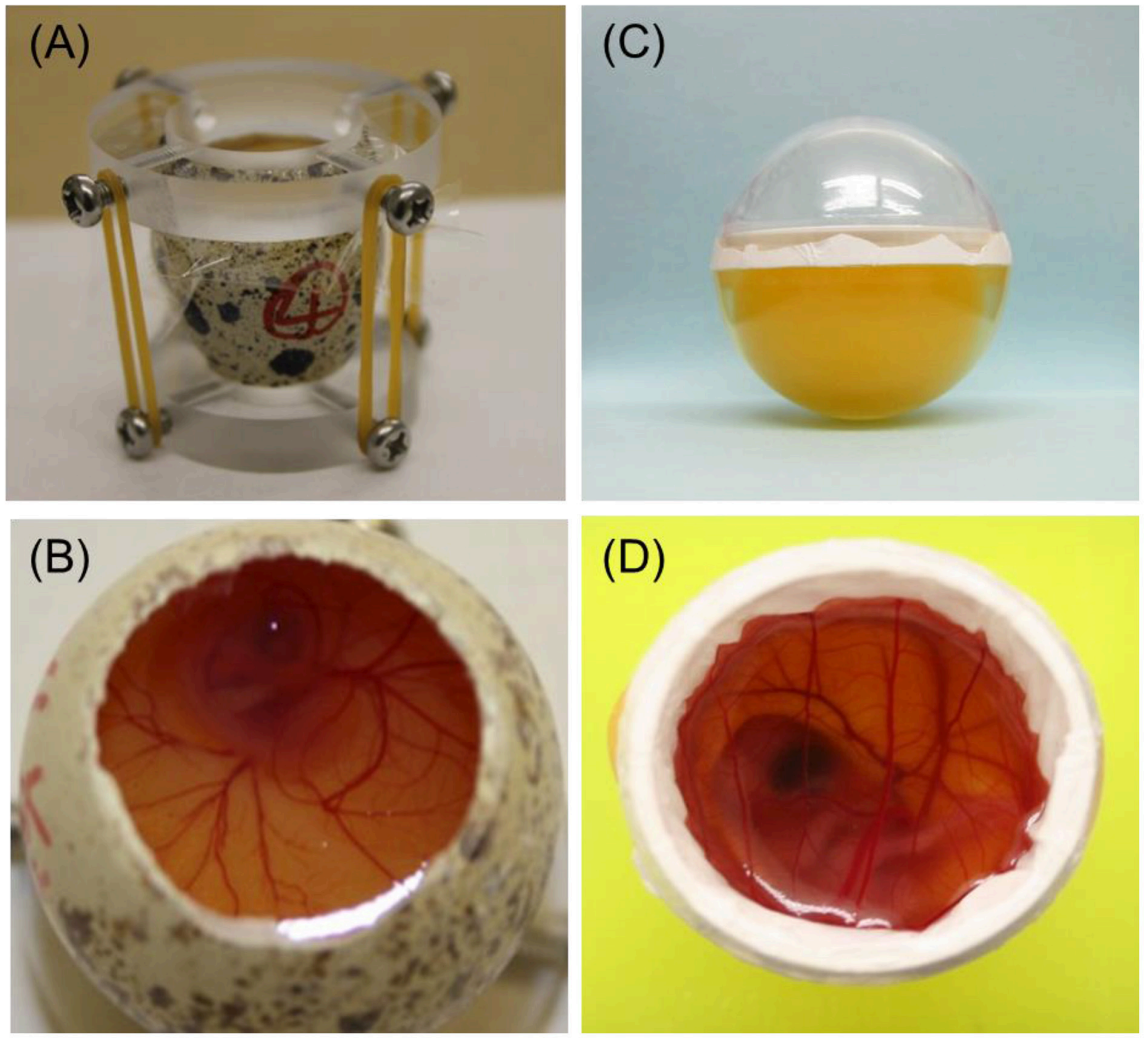
**Photographs of two quail embryo culture systems (ECSs). (A)** Perry's ECS (System II) uses a surrogate eggshell for the period from blastoderm to embryogenesis. **(B)** Perry's ECS (System III) uses a surrogate eggshell during embryonic growth until hatching. **(C)** Alternative ECS using an artificial culture vessel for the period from blastoderm to embryogenesis. Developing quail embryos can be observed by tipping the vessel slightly, allowing easy determination of the developmental stage. The volumes of the lower halves of the plastic cases (diameter across the equatorial plane, 26 mm) were comparable with those of quail eggs. **(D)** Alternative ECS using an artificial culture vessel during embryonic growth until hatching.

The first avian whole ECS was established in chicken by Perry (1988) and developed further by Naito et al. (1990). An ECS has also been developed for Japanese quail (Ono et al., 1994). Perry's ECS (1988) covers the period from fertilization of the ovum to hatching and comprises three culture systems (Perry, 1988). System I is used to culture embryos obtained from the oviduct in the early cleavage stages, that is, to culture embryos from fertilization to blastoderm formation (Phase I). Systems II (Figure 2A) and III (Figure 2B) use a surrogate eggshell to culture embryos obtained from newly laid eggs through the period of embryogenesis (Phase II) and from embryonic growth to hatching (Phase III).

Our group has also succeeded in cultivating newly laid chicken or quail eggs through the period of embryogenesis (Phase II) by using a clear, egg-shaped artificial culture vessel (Figure 2C), which allowed direct observation of the developmental stage at any time throughout the cultivation period simply by tipping the vessel (Kawashima et al., 2005). In a comparison of our ECS, which uses an artificial culture vessel, and Perry's System II culture system, which uses a surrogate eggshell culture vessel, no obvious detrimental effects were found in Phase II embryos cultured by using our shell-less culture method (Kawashima et al., 2005).

Attempts to culture avian embryos *in vitro* from embryonic growth to hatching (i.e., Phase III) have also been made. Auerbach et al. (1974) cultured 3- to 4-day-old chicken embryos in Petri dishes and obtained approximately 40% viability at 14 days of total incubation with a maximum development period of 18 days. Dunn and Boone (1976) cultured 3-day-old chicken embryos in egg-shaped plastic wrap and reported that the maximum developmental stage reached was almost before hatching. Ono and Wakasugi (1983) cultured quail embryos, preincubated for 2.5 days, by using a plastic wrap technique, but the embryos did not survive beyond 14 days. Finally, Kamihira et al. (1998) succeeded in culturing 2-day-old quail embryos to hatching by using a gas-permeable Teflon membrane (Milliwrap; Nihon Millipore Co., Tokyo, Japan) with calcium supplementation and oxygen aeration (Figure 2D). Therefore, it is now possible to cultivate avian embryos in an artificial vessel throughout the whole embryonic growth phase.

Avian-based toxicity screening tests that are simple to conduct, rapid, and cost-effective are urgently required to obtain preliminary information on the effects of exposure to endocrine-disrupting chemicals in vertebrates (Flint et al., 2012; Oshima et al., 2012). An avian *in ovo* model called the sex reversal test has been established that uses Japanese quail embryos (Shibuya et al., 2004). Histologically, intact male Japanese quail embryos in the later embryonic stages just before hatching develop both left and right testes, whereas intact female embryos develop only a left-side ovary. Exposure of the embryo to estrogen or to an estrogen-like chemical such as an endocrine-disrupting chemical during an early embryonic developmental stage causes transformation of the left testis into an ovotestis and persistence of the left oviduct in genetic males (Shibuya et al., 2005). Therefore, the sex reversal test using an avian ECS represents a high-throughput, quantitative approach for the evaluation of the estrogenic effects of endocrine-disrupting or other chemicals.

Avian ECSs offer ethical advantages over other *in vivo* means of toxicity screening, especially when developmental toxicity of a chemical is anticipated. Furthermore, avian ECSs offer the additional advantage of allowing direct quantitative determination of the effects of test chemicals on the fetus without any maternal influence through placental transmission. Furthermore, because avian ECSs facilitate direct observation and manipulation, it is now possible to investigate the time-specific influences of chemicals with embryonic toxicity on the embryo. Suitable ECSs should be developed depending on the research or testing goal.

## Features of avian neurobehavior

Avian experimental models may also be useful for assessing aspects of neurobehavior such as the sociosexual behaviors of aggression and mating using Japanese quail (Ubuka et al., 2013). Indeed, some avian species have already been used as models of learning and memory in studies examining the neural basis of cognition (Emery, 2006). Corvids, songbirds, and domestic fowl are currently considered the best models available to examine specific aspects of the neurobiology underlying learning and cognition (Clayton and Emery, 2015). To apply the usefulness of avian models for learning and cognition to the developmental toxicity research, we need to establish new endpoints appropriate for the evaluation of neurotoxic and neurobehavioral effects.

Japanese quails have been used as a model organism in studies of the neurophysiological and neuroendocrine bases of aggression and reproductive behavior for a long period (Selinger and Bermant, 1967; Mills et al., 1997; Ubuka et al., 2013; Ubuka and Tsutsui, 2014). Sexually mature male quail frequently fight with intense aggression and display a series of stereotypical actions. They often approach, chase, and peck their opponent (peck), grab the back of their opponent's head or neck with their beak (grab), attempt to ride on the back of their opponent (mount), or ride on the back of their opponent and lower their cloaca close to their opponent's cloaca (cloacal contact-like action). Since the behavior of each quail can be recorded and analyzed by using a digital video camera, the numbers of pecks, grabs, mounts, and cloacal contact-like actions attempted by each animal can be quantified (see in Supplementary Movie 1). Since the frequency of these actions represents the degree of the sociosexual behavior of individual male quails (Ubuka et al., 2013), these actions represent novel endpoints that could be used to assess developmental toxicity.

Imprinting, which occurs limitedly during the initial stage of avian chicks, may be useful to establish a behavioral index for investigating the neural plasticity involved in juvenile learning (Yamaguchi et al., 2012; Nakamori et al., 2013). Imprinting is characterized by a high learning efficiency and robust memory retention, which are features that distinguish it from general learning and memory. Maekawa et al. (2006) established an experimental procedure for imprinting to visual stimuli presented on a liquid crystal display. After training, the chicks are placed on a running wheel, and an angle sensor is used to record the number of forward and backward rotations of the wheel as the chick is shown either images they had been shown since hatching or new, previously unseen images. The effects of imprinting on neural activity in the visual wulst can also be investigated by using *in vivo* intrinsic optical imaging techniques (Maekawa et al., 2007). It may also be possible to apply a measurement system of juvenile learning and memory that utilizes avian imprinting behavior as a novel means of rapidly assessing developmental neurotoxicity in early childhood.

Imprinting in Japanese quail has also been reported. It has been found that Japanese quail will imprint on achromatic stimuli, flashing lights, and models of quail hens (Mills et al., 1997). Imprinting in chicks may be a good model of learning in human infants because human infants and chicks share a critical period during which they are sensitive to specific experiences. Therefore, uncovering the molecular mechanisms underlying the imprinting process will help clarify the juvenile learning, which has an obvious critical period (Suzuki et al., 2012). Since future studies are expected to reveal the molecular mechanisms underlying learning and memory in higher vertebrates, new developmental neurotoxicity endpoints are also likely to be established in the future.

## Can avian test battery be alternative to developmental neurotoxicity tests for human?

The combination of an avian ECS followed by neurobehavioral assessment is also a promising means of assessing developmental neurotoxicity. For many chemicals, it is high prenatal exposure that induces postnatal neurobehavioral disorders (Grandjean and Landrigan, 2006; Huizink and Mulder, 2006). However, even if exposure to a certain chemical is not found to induce apparently morphological abnormalities in the fetal and infantile brain, the chemical may still cause functional disorders and affect learning and memory. By using brain functions such as behavior, memory, and learning as advanced endpoints, it may be possible to use avian-based assay systems in place of the currently used tests of developmental neurotoxicity. However, given that the morphological development of the brain may differ between humans and birds, further studies are needed to determine how best to extrapolate test results.

Most of the currently used toxicity studies for environmental chemicals use acute or chronic systemic or reproductive toxicity endpoints and use mammalian or avian laboratory animals. However, since exposure in mammalian models is usually based on how much chemical is administered to the mother, it is difficult to determine exactly how much the fetus receives through the placenta. The Organization for Economic Cooperation and Development has established a series of standardized ecological risk assessments for chemicals such as endocrine disruptors (Buschmann, 2013). Although, these tests are accepted internationally as standard methods for evaluating the risk to human health and wildlife posed by persistent and degradable chemicals in the environment, neurobehavioral endpoints of toxicity are rarely examined. In addition, given the increasing number of new chemicals that potentially have neurotoxic effects in vertebrates, including humans, a rapid means of assessing the neurotoxic effects of these chemicals in humans is required.

Recently, it has been suggested that neonicotinoid pesticides have caused a decline in wild bird populations (Hallmann et al., 2014; Gibbons et al., 2015). Since the use of neonicotinoid pesticides is rapidly increasing throughout the world, the effects of these pesticides on human health are now a concern. Even if neonicotinoid pesticides were found not to adversely affect the morphological development of the embryonic human brain, the question of whether or not they cause postnatal neurobehavioral disorders would remain. Therefore, there is an urgent need to establish animal-based evaluation systems that can be used to assess neurotoxic effects of neonicotinoid pesticides. The avian test battery represents a potentially rapid and cost-effective way of conducting these assays.

## Conclusion

Avian test batteries represent a potentially rapid, cost-effective, ethical alternative to the currently available means of assessing developmental toxicity using higher vertebrates. Here we have discussed using a Galliformes-based avian test battery in which developmental toxicity is assessed by means of a combination of chemical exposure during early embryonic development by using an ECS followed by analyses in later life of sociosexual behaviors such as aggression and mating and of visual memory via filial imprinting. However, to fully harness the potential of this novel means of chemical toxicity screening, it will be necessary to establish a variety of evaluation systems and experimental models before we can extrapolate the test results to humans.

## Author contributions

All authors contributed to substantial contributions to the conception or design of the work. TK, WA, and KN designed this work. TK, TU, and KT revised the important intellectual content critically. TK wrote the article and all authors contributed to the editing.

### Conflict of interest statement

The authors declare that the research was conducted in the absence of any commercial or financial relationships that could be construed as a potential conflict of interest.

## Supplementary material

Supplementary Movie 1 **Series of stereotyped aggressive and reproductive behaviors in masculine Japanese quail, namely, pecks, grabs, mounts, and cloacal contact-like actions**.
